# Discriminatory ability of simple OGTT-based beta cell function indices for prediction of prediabetes and type 2 diabetes: the CODAM study

**DOI:** 10.1007/s00125-016-4165-3

**Published:** 2016-12-08

**Authors:** Louise J. C. J. den Biggelaar, Simone J. S. Sep, Simone J. P. M. Eussen, Andrea Mari, Ele Ferrannini, Marleen M. J. van Greevenbroek, Carla J. H. van der Kallen, Casper G. Schalkwijk, Coen D. A. Stehouwer, Pieter C. Dagnelie

**Affiliations:** 1 Department of Epidemiology, Maastricht University, PO Box 616, 6200 MD Maastricht, the Netherlands; 2 School for Cardiovascular Diseases (CARIM), Maastricht University, Maastricht, the Netherlands; 3 Department of Internal Medicine, Maastricht University Medical Center, Maastricht, the Netherlands; 4 School for Public Health and Primary Care (CAPHRI), Maastricht University, Maastricht, the Netherlands; 5 CNR Institute of Neurosciences, Padua, Italy; 6 CNR Institute of Clinical Physiology, Pisa, Italy

**Keywords:** Beta cell function, Discrimination, Indices, Insulin secretion, OGTT, Prediabetes, Receiver operating characteristics, Type 2 diabetes mellitus

## Abstract

**Aims/hypothesis:**

The hyperglycaemic clamp technique and the frequently sampled IVGTT are unsuitable techniques to assess beta cell function (BCF) in large cohorts. Therefore, the aim of this study was to evaluate the discriminatory ability of simple OGTT-based BCF indices for prediction of prediabetes (meaning impaired fasting glucose and/or impaired glucose tolerance) and type 2 diabetes.

**Methods:**

Glucose metabolism status was assessed by 2 h 75 g OGTT at baseline (*n* = 476, mean age 59.2 years, 38.7% women) and after 7 years of follow-up (*n* = 416) in the Cohort on Diabetes and Atherosclerosis Maastricht (CODAM) study (1999–2009). Baseline plasma glucose, insulin and C-peptide values during OGTTs were used to calculate 21 simple indices of BCF. Disposition indices (BCF index × Matsuda index), to compensate for the prevailing level of insulin resistance, were calculated for the BCF indices with the best discriminatory abilities. The discriminatory ability of the BCF indices was estimated by the area under the receiver operating characteristics curve (ROC AUC) with an outcome of incident prediabetes (*n* = 73) or type 2 diabetes (*n* = 60 and *n* = 18 cases, respectively, in individuals who were non-diabetic or had normal glucose metabolism at baseline).

**Results:**

For incident prediabetes (*n* = 73), all ROC AUCs were less than 70%, whereas for incident type 2 diabetes, I_30_/I_0_, CP_30_/CP_0_, ΔI_30_/ΔG_30_, ΔCP_30_/ΔG_30_ (where I, CP and G are the plasma concentrations of insulin, C-peptide and glucose, respectively, at the times indicated), and corrected insulin response at 30 min had ROC AUCs over 70%. In at-baseline non-diabetic individuals, disposition indices ΔI_30_/ΔG_30_, ΔCP_30_/ΔG_30_ and corrected insulin response at 30 min had ROC AUCs of over 80% for incident type 2 diabetes. Moreover, these BCF disposition indices had significantly better discriminatory abilities for incident type 2 diabetes than the Matsuda index alone.

**Conclusions/interpretation:**

BCF indices reflecting early-phase insulin secretion have the best ability to discriminate individuals who will develop prediabetes and type 2 diabetes. Of these, ΔCP_30_/ΔG_30_, often referred to as the C-peptidogenic index, performed consistently well.

**Electronic supplementary material:**

The online version of this article (doi:10.1007/s00125-016-4165-3) contains peer-reviewed but unedited supplementary material, which is available to authorised users.

## Introduction

Type 2 diabetes is characterised by reduced metabolic sensitivity to insulin and insufficient insulin secretion by pancreatic beta cells. Insulin resistance in itself is an insufficient cause of type 2 diabetes, because of the pancreatic beta cells’ ability to compensate for the prevailing level of insulin resistance by increasing insulin secretion. However, if the beta cells are unable to compensate for the prevailing insulin resistance, hyperglycaemia arises and can cause a transition from NGM to prediabetes, and eventually to type 2 diabetes [1–3].

The assessment of beta cell function (BCF) in vivo is complex because of its interplay with other key variables in glucose homeostasis, i.e. blood glucose levels, insulin sensitivity and hepatic insulin extraction [2, 4]. The simplest methods for the assessment of BCF in epidemiological studies are those based on basal circulating levels of glucose, insulin and/or C-peptide, such as HOMA-B [5]. Fasting levels, however, cannot provide insight into the secretory response of beta cells to rising and falling glucose concentrations [2, 3, 6]. Alternative approaches are based on beta cell responses after administration of nutrients, often glucose. Intravenous approaches include the hyperglycaemic clamp technique and the frequently sampled IVGTT. However, disadvantages of these methods are complex protocols, high costs and the non-physiological route and pattern of glucose administration [3, 6].

Oral administration of nutrients, as applied during an OGTT or mixed-meal tolerance test, enables assessment of the insulin secretory response under more physiological conditions [6]. Both oral methods stimulate the incretin hormone effect and allow for the simultaneous assessment of BCF, insulin sensitivity and glucose metabolism status. The mixed-meal tolerance test most closely tracks the physiological responses that are expected to occur during an individual’s normal day-to-day life (to a load of mixed nutrients). The OGTT, however, can be standardised more easily and is simple when it comes to administering the glucose load. Therefore, the OGTT is often the preferred method in large epidemiological studies.

Several simple BCF indices obtained from an OGTT have been proposed and validated against intravenous methods, with the major drawback of a non-physiologically triggered insulin secretory response in the latter. Therefore, the objective of this study was to compare available, simple OGTT-based BCF indices that use plasma concentrations of glucose, insulin and/or C-peptide to identify their ability to discriminate glucose metabolism status (normal glucose metabolism [NGM], prediabetes [meaning impaired fasting glucose and/or impaired glucose tolerance] and type 2 diabetes), which may approximate conceptually a more physiological aspect of BCF assessment.

## Methods

### Study population and design

We used data from the Cohort on Diabetes and Atherosclerosis Maastricht (CODAM) study, a longitudinal observational study on the natural progression of glucose tolerance [7]. CODAM includes 574 individuals with an elevated risk of type 2 diabetes and cardiovascular disease [8], who were extensively characterised at baseline with regard to their lifestyle and cardiovascular and metabolic profile during two visits to the university’s metabolic research unit. After a median follow-up period of 7.0 years (interquartile range [IQR] 6.9–7.1 years), 491 individuals participated in the follow-up measurements (overall attrition rate 14%). The participants who withdrew from the study during follow-up had essentially the same baseline characteristics as the study cohort. The CODAM study was approved by the Medical Ethical Committee of the Maastricht University Medical Center, and all participants gave written informed consent.

For the present analyses, we excluded 83 individuals with previously diagnosed type 2 diabetes at baseline (defined as self-reported diagnosis and/or use of glucose-lowering medication) who did not undergo an OGTT as per the protocol, and 15 individuals because of incomplete OGTT data. This resulted in a study population of 476 individuals, with glucose metabolism ranging from NGM (*n* = 294) to prediabetes (*n* = 122) to newly diagnosed type 2 diabetes (*n* = 60), using the WHO criteria as specified below [9]. Longitudinal analyses were restricted to individuals without type 2 diabetes who participated in the follow-up measurements (*n* = 294 NGM, *n* = 122 prediabetes), of whom 73 (24.8%) individuals with NGM progressed to prediabetes, and 17 (5.8%) individuals with NGM and 46 (37.7%) with prediabetes progressed to type 2 diabetes.

### OGTT and glucose metabolism status

After an overnight fast (>12 h), venous blood samples were collected before and 30, 60 and 120 min after ingestion of 75 g glucose. Plasma for the assessment of insulin and C-peptide was collected in EDTA tubes on ice, separated after centrifugation (3000×*g* for 15 min at 4°C), and stored at −80°C until the assays were performed. The time between collection and storage was less than 2 h.

Insulin and C-peptide were measured by use of a custom Meso Scale Discovery duplex array (Meso Scale Discovery, Gaithersburg, MD, USA; www.mesoscale.com). In short, 96 well-plates, with capture antibodies against insulin and C-peptide patterned on distinct spots in the same well were supplied by the manufacturer. Samples (10 μl/well), detection antibodies and read buffer for electrochemiluminescence were applied according to manufacturer’s instruction, and plates were read using a SECTOR Imager 2400 (Meso Scale Discovery). The detection ranges of the assay were 35–25,000 pg/ml for insulin and 70–50,000 pg/ml for C-peptide. Interassay CVs for insulin and C-peptide were 9.7% and 7.9%, respectively. Insulin and C-peptide values were converted from pg/ml to pmol/l using a molar mass of 5808 g for insulin and 3010 g for C-peptide. Plasma for the assessment of glucose was collected in NaF/KOx tubes on ice. Glucose was measured by use of the hexokinase method (HK-G6PD method; Glucose HK 125; ABX Diagnostics, Montpellier, France).

Glucose metabolism status was defined according to the WHO 2006 criteria [9]: NGM (fasting plasma glucose <6.1 mmol/l and 2 h post-load plasma glucose <7.8 mmol/l), prediabetes (fasting plasma glucose levels of 6.1–6.9 mmol/l and/or 2 h post-load glucose levels of 7.8–11.1 mmol/l) and type 2 diabetes (fasting plasma glucose ≥7.0 mmol/l and/or 2 h post-load glucose ≥11.1 mmol/l).

### OGTT-derived measures of BCF

Available OGTT-based BCF indices were extracted from the literature and included nine early-phase measures (based on the first 30 min of the OGTT), six late-phase measures (based on the fasting condition combined with the last 60–120 min of the OGTT) and two overall insulin secretion measures (based on all OGTT sampling points) (Table 1).

**Table 1:** OGTT-based indices of BCF

BCF index	Literature reference	Calculation
Fasting indices		
HOMA-B1 (%)	[5]	(20 × I_0_)/(G_0_ - 3.5)
HOMA-B2 (%)	[32]	Web-based calculator using the HOMA2 model [33]
Insulin:glucose ratio t_0_	[34]	I_0_/G_0_
C-peptide:glucose ratio t_0_	[35]	CP_0_/G_0_
Early-phase indices		
Insulin ratio t_30_	[36]	I_30_/I_0_
C-peptide ratio t_30_	[36]	CP_30_/CP_0_
Insulinogenic index t_30_	[35]	(I_30_ - I_0_)/(G_30_ - G_0_)
Modified insulinogenic index t_30_	[37]	(I_30_ - I_0_)/(G_30_)
C-peptidogenic index t_30_	[35]	(CP_30_ - CP_0_)/(G_30_ - G_0_)
CIR_30_ × 10^−2^	[38]	I_30_/[G_30_ × (G_30_ - 3.89)]
Stumvoll early-phase × 10^−2^	[39]	1283 + (1.829 × I_30_) - (138.7 × G_30_) + (3.772 × I_0_)
BIGTT-AIR_0.30.120_ × 10^−2^	[40]	Exp[8.20 + (0.00178 × I_0_) + (0.00168 × I_30_ ) - (0.000383 × I_120_) - (0.314 × G_0_) - (0.109 × G_30_) + (0.0781 × G_120_) + (0.180 × sex) - (0.032 × BMI)]
BIGTT-AIR_0.60.120_ × 10^−2^	[40]	Exp[8.19 + (0.00339 × I_0_) + (0.00152 × I_60_) - (0.000959 × I_120_) - (0.389 × G_0_) - (0.142 × G_60_) + (0.164 × G_120_) + (0.256 × sex) + (0.038 × BMI)]
Late-phase indices		
Stumvoll second phase	[39]	287 + (0.4164 × I_30_) - (26.07 × G_30_) + (0.9226 × I_0_)
Insulin ratio t_120_	[36]	I_120_/I_0_
C-peptide ratio t_120_	[36]	CP_120_/CP_0_
Insulinogenic index t_120_	[35]	(I_120_ - I_0_)/(G_120_ - G_0_)
C-peptidogenic index t_120_	[35]	(CP_120_ - CP_0_)/(G_120_ - G_0_)
CIR_120_ × 10^−2^	[38]	I_120_/(G_120_ × (G_120_ - 3.89))
Overall indices		
AUC insulin:glucose ratio	[41]	[(30 - 0) × (I_0_ + I_30_)/2] + [(60 - 30) × ( I_30_ + I_60_) / 2] + [(120 - 60) × ( I_60_ × I_120_)/2)] / [(30 - 0) × (G_0_ + G_30_) / 2] + [(60 - 30) × ( G_30_ + G_60_) / 2] + [(120 - 60) × ( G_60_ × G_120_) / 2]
AUC C-peptide:glucose ratio	[41]	[(30 - 0) × (CP_0_ + CP_30_) /2] + [(60 - 30) × (CP_30_ + CP_60_) / 2] + [(120 - 60) × (CP_60_ × CP_120_) /2)] / [(30 - 0) × (G_0_ + G_30_) / 2] + [(60 - 30) × (G_30_ + G_60_) / 2) + ((120 - 60) × (G_60_ × G_120_) / 2)]

BIGTT-AIR, beta cell function, insulin sensitivity and glucose tolerance testing; t, time point during OGTT, min in subscript

Kahn et al identified a hyperbolic relationship between insulin sensitivity and secretion [10]. The product of insulin sensitivity and insulin secretion is called the disposition index (DI) and reflects a measure of BCF corrected for the degree of insulin sensitivity. In this study, the Matsuda index was used as a measure of insulin sensitivity (Matsuda index: 10,000 / √G_0_ × I_0_ × mean G × mean I; where G and I are the plasma glucose and insulin values, respectively, at the time indicated) [11].

The OGTT-based mathematical model of Mari et al [12] describes insulin secretion as the sum of two components. Glucose sensitivity describes the degree of pancreatic beta cell responsiveness to absolute blood glucose levels (glucose–insulin dose–response curve). The beta cell potentiation factor modulates the dose–response curve as a positive function of time in individuals with NGM. The second component of insulin secretion is beta cell rate sensitivity, which represents early-phase insulin release. We evaluated these mathematical model-based BCF indices as additional analyses as these BCF components cannot be assessed by simple BCF indices.

### Study population characteristics

Body height (cm) and weight (kg) were measured to the nearest 1 cm and 0.1 kg with the participants wearing light clothing and no shoes [7]. Total cholesterol, HDL-cholesterol and triacylglycerol were determined using enzymatic techniques (Roche Diagnostics, Mannheim, Germany). LDL-cholesterol was calculated using the Friedewald formula [13]. NEFA were assessed in EDTA plasma using an enzymatic colorimetric method (NEFA-C; Wako Chemicals, Neuss, Germany) [14]. Blood pressure was measured using an oscillometric precision blood pressure instrument (Maxi-Stabol 3; Speidel & Keller, now Welch Allyn, Skaneateles Falls, NY, USA) [15]. Smoking status, medication use and the prevalence of a history of cardiovascular disease and cardiovascular events were determined by self-report [16]. Physical activity was measured by the validated Short Questionnaire to Assess Health-enhancing Physical Activity (SQUASH) [17].

### Statistical methods

All analyses were performed using the software package SPSS statistics version 22.0 for Windows (SPSS, IBM Corp, Armonk, NY, USA). Differences in median BCF across glucose metabolism groups were tested by Kruskal–Wallis and Mann–Whitney *U* tests.

The primary outcome was the discriminatory ability of the BCF indices to predict prediabetes and type 2 diabetes. The discriminatory ability was assessed by use of the area under the receiver operating characteristics (ROC) curve with either non-prediabetes (i.e. NGM) or non-diabetes as the reference category. Differences between consecutively ranked ROC AUCs were tested by the algorithm developed by DeLong et al [18]. ROC AUCs of the DIs (the products of the BCF index and Matsuda index) were assessed for BCF indices with ROC AUCs of 0.70 or above for incident prediabetes and/or type 2 diabetes [19].

Additional analyses were performed to cross-sectionally evaluate the discriminatory abilities of the BCF indices for prevalent prediabetes and type 2 diabetes. Finally, the discriminatory ability of the mathematical model variables of Mari et al [12] were evaluated: beta cell glucose sensitivity, beta cell rate sensitivity and beta cell potentiation factor.

The confidence level used in the statistical analyses was 95%, corresponding to a *p* value of 0.05.

## Results

The median age was similar across groups of glucose metabolism status at baseline (Table 2). The male to female ratio was higher in participants with type 2 diabetes. Median fasting and 2 h post-load plasma concentrations of glucose and insulin, and HbA_1c_ increased in the order NGM to prediabetes to type 2 diabetes, whereas median insulin sensitivity decreased with impairment of glucose metabolism. Furthermore, BP increased and the lipid profile worsened with impairment of glucose metabolism. In addition, the proportion of current smokers was lowest among individuals with type 2 diabetes, but individuals with prediabetes or type 2 diabetes were less physically active than individuals with NGM (Table 2).

**Table 2:** Baseline characteristics: individuals with NGM, prediabetes and newly diagnosed type 2 diabetes mellitus (T2DM)

Variable	NGM *n* = 294	Prediabetes *n* = 122	T2DM *n* = 60
Age (years)	59.7 (53.0, 64.2)	60.8 (55.3, 64.9)	60.1 (56.3, 64.1)
Women, *n* (%)	120 (40.8)	47 (38.5)	17 (27.9)
BMI (kg/m^2^)	27.3 (25.0, 29.5)	28.0 (26.5, 31.2)	30.6 (26.6, 32.8)
Current smoker, *n* (%)	60 (20.4)	22 (18.0)	9 (14.8)
Physical activity (10^3^ × METs/week)	6.54 (3.94, 8.94)	5.10 (2.79, 8.46)	5.64 (3.73, 7.78)
Systolic BP (mmHg)	133 (122, 146)	141 (132, 155)	145 (136, 158)
Diastolic BP (mmHg)	80.0 (73.5, 85.0)	82.8 (78.0, 91.5)	85.0 (79.5, 92.8)
Antihypertensive medication, *n* (%)	82 (27.9)	52 (42.6)	29 (47.5)
CVD, *n* (%)	68 (23.1)	34 (28.1)	24 (39.3)
CVE, *n* (%)	40 (13.6)	20 (16.5)	11 (18.0)
Total cholesterol (mmol/l)	5.20 (4.60, 5.80)	5.20 (4.60, 5.83)	5.50 (4.90, 6.00)
HDL-cholesterol (mmol/l)	1.23 (1.01, 1.44)	1.09 (0.93, 1.37)	0.97 (0.86, 1.20)
LDL-cholesterol (mmol/l)	3.30 (2.80, 3.90)	3.35 (2.80, 4.30)	3.50 (2.90, 3.98)
HDL:LDL ratio	0.37 (0.28, 0.48)	0.32 (0.26, 0.45)	0.30 (0.24, 0.37)
Triacylglycerol (mmol/l)	1.20 (0.90, 1.60)	1.60 (1.10, 2.10)	1.90 (1.30, 2.65)
NEFA (mmol/l)	0.49 (0.38, 0.57)	0.53 (0.43, 0.64)	0.55 (0.45, 0.70)
Lipid-lowering medication, *n* (%)	44 (15.0)	24 (19.7)	9 (14.8)
HbA_1c_ (%)	5.70 (5.40, 5.90)	5.80 (5.60, 6.10)	6.40 (5.90, 6.90)
HbA_1c_ (mmol/mol)	38.0 (35.0, 40.0)	39.0 (37.0, 43.0)	46.0 (40.0, 51.0)
Fasting glucose (mmol/l)	5.27 (5.00, 5.53)	6.00 (5.54, 6.30)	7.14 (6.81, 7.95)
Fasting insulin (pmol/l)	61.2 (45.2, 87.4)	78.8 (51.4, 133)	110 (66.1, 159)
2 h glucose (mmol/l)	5.65 (4.65, 6.54)	8.79 (7.82, 9.90)	12.3 (11.1, 14.7)
2 h insulin (pmol/l)	352 (211, 589)	664 (424, 1176)	744 (489, 1017)
Matsuda index of insulin sensitivity	3.57 (2.45, 4.95)	2.46 (1.39, 3.63)	1.45 (1.09, 2.66)

Values are expressed as median (IQR) or *n* (%)

CVD, self-reported cardiovascular disease; CVE, self-reported cardiovascular event; MET, metabolic equivalent

Overall, the median values of all BCF indices differed statistically significantly by glucose metabolism status at baseline, reflecting the fact that impaired BCF went along with impairment of glucose metabolism (Table 3). For most indices, the largest proportional difference in BCF was observed between individuals with prediabetes and type 2 diabetes.

**Table 3:** Values of BCF indices according to glucose metabolism status at baseline

BCF index	NGM *n* = 294	IGM *n* = 122	T2DM *n* = 60	*p* values^a^
Fasting indices				
HOMA-B1 (%)	105.39 (74.60, 144.20)	99.78 (64.62, 169.02)	88.58 (53.25, 118.20)^†^	0.037
HOMA-B2 (%)	96.90 (76.70, 119.35)	92.00 (67.20, 133.83) *	79.40 (55.75, 98.25)^†^	0.001
I_0_/G_0_ ratio^b^	11.45 (8.55, 16.4)	13.21 (8.58, 13.13)	15.82 (9.69, 21.22)^†§^	0.007
CP_0_/G_0_ ratio^b^	0.12 (0.95, 0.15)	0.13 (0.10, 0.18)	0.14 (0.12, 0.17)^†§^	0.016
Early-phase BCF indices				
I_30_/I_0_ ratio	7.98 (6.14, 10.4)	5.81 (4.61, 7.42)*	3.62 (2.74, 3.62)^†§^	<0.001
CP_30_/CP_0_ ratio	3.38 (2.91, 4.22)	2.68 (2.25, 3.30)*	1.96 (1.74, 2.38)^†§^	<0.001
ΔI_30_/ΔG_30_ ratio	135.50 (88.27, 198.93)*	90.75 (53.81, 151.89)*	58.02 (38.11, 85.16)^†§^	<0.001
ΔCP_30_/ΔG_30_ ratio	0.49 (0.33, 0.66)	0.30 (0.23, 0.42)*	0.19 (0.15, 0.26)^†§^	<0.001
CIR_30_ × 10^−2^	12.91 (8.29, 19.15)	8.02 (4.34, 11.54)*	3.68 (2.72, 5.86)^†§^	<0.001
Stumvoll first phase × 10^−2^	12.53 (9.33, 16.76)	10.83 (6.08, 17.51)*	7.08 (4.08, 12.56)^†§^	<0.001
BIGTT-AIR_0.30.120_ × 10^−2^	29.64 (21.98, 43.84)	24.11 (15.36, 37.02)*	17.33 (11.77, 24.90)^†§^	<0.001
BIGTT-AIR_0.60.120_ × 10^−2^	34.00 (25.81, 51.12)	27.32 (17.08, 44.66)*	19.42 (12.37, 31.24)^†§^	<0.001
Late-phase BCF indices				
Stumvoll second phase	330.71 (258.45, 431.44)	310.64 (187.11, 457.80)*	239.06 (152.97, 358.97)^†^	<0.001
I_120_/I_0_ ratio	5.60 (3.77, 8.27)	8.42 (5.94, 11.7)*	6.85 (4.32, 10.37)^†§^	<0.001
CP_120_/CP_0_ ratio	4.04 (3.21, 5.08)	4.51 (3.64, 5.60)	3.73 (2.59, 5.31)^§^	<0.001
ΔI_120_/ΔG_120_ ratio	235.21 (, 122.49, 532.01)	200.50 (113.36, 403.98)*	114.48 (66.54, 178.28)	0.037
ΔCP_120_/ΔG_120_ ratio	1.35 (, 1.29, 2.83)	0.91 (0.59, 1.32)*	0.47 (0.31, 0.67)^†^	0.009
CIR_120_ × 10^−2^	35.21 (21.38, 60.71)	17.45 (11.13, 31.92)*	7.29 (4.42, 7.29)^†§^	<0.001
Overall indices				
I_AUC_ / G_AUC_ ratio	62.03 (45.50, 86.60)	56.71 (35.91, 93.40)*	42.27 (26.70, 63.07)^†^	<0.001
CP_AUC_/ G_AUC_ ratio	0.35 (0.26, 0.38)	0.28 (0.21, 0.37)*	0.20 (0.16, 0.24)^†^	<0.001

Values are expressed as median (IQR)

^a^ *p* value of Kruskal–Wallis test

^b^A higher value indicates impairment of BCF, unlike other BCF indices

*Statistically significant difference IGM vs NGM, *p* value of Mann–Whitney *U* test <0.05; ^†^statistically significant difference T2DM vs NGM, *p* value of Mann–Whitney *U* test <0.05; ^§^statistically significant difference T2DM vs IGM, *p* value of Mann–Whitney *U* test <0.05

BIGTT-AIR, beta cell function, insulin sensitivity and glucose tolerance testing; T2DM, type 2 diabetes mellitus

### Incident prediabetes and type 2 diabetes

Of the individuals with NGM or prediabetes at baseline, 63 (17 NGM and 46 prediabetes) progressed to type 2 diabetes at follow-up, and 73 individuals with NGM progressed to prediabetes at follow-up (Table 4).

**Table 4:** Discrimination between individuals with incident type 2 diabetes mellitus (T2DM) or prediabetes in individuals who were NGM or non-T2DM at baseline

BCF index	Incident T2DM in at-baseline Non-T2DM Follow-up *n* = 306 non-T2DM *n* = 63 T2DM	Rank	Incident T2DM in at-baseline NGM Follow-up *n* = 17 T2DM *n* = 170 NGM	Rank	Incident prediabetes in at-baseline NGM Follow-up *n* = 73 prediabetes *n* = 170 NGM	Rank
Fasting measures						
HOMA-B1	52 (44, 61)	20	64 (48, 81)	9	54 (46, 62)	15
HOMA-B2	52 (43, 60)	21	65 (48, 82)	8	53 (45, 62)	16
I_0_/G_0_ ratio	59 (51, 67)	16	69 (54, 85)	7	51 (43, 60)	20
CP_0_/G_0_ ratio	60 (52, 68)	14	70 (55, 84)	6	52 (44, 60)	19
Early-phase BCF indices						
I_30_/I_0_ ratio	77 (70, 83)	4	72 (60, 84)	5	55 (47, 63)	9–11
CP_30_/CP_0_ ratio	78 (72, 84)	2	76 (65, 87)	3	50 (43, 58)	21
ΔI_30_/ΔG_30_ ratio	74 (68, 81)	5	73 (61, 85)	4	59 (51, 67)	5
ΔI_30_/G_30_ ratio	69 (62, 77)	6	63 (49, 77)	10	55 (47, 63)	9–11
ΔCP_30_/ΔG_30_ ratio	81 (75, 86)	1	84 (75, 93)	1	62 (54, 70)	2–3
CIR_30_	77 (71, 83)	3	77 (67, 87)	2	61 (53, 68)	4
Stumvoll early-phase × 10^−2^	62 (54, 71)	10	52 (36, 69)	18	55 (47, 63)	9–11
BIGTT_0.30.120_ × 10^−2^	63 (55, 71)	9	58 (42, 73)	12	54 (46, 63)	13–14
BIGTT_0.60.120_ × 10^−2^	61 (52, 69)	12	51 (33, 69)	20	57 (49, 65)	6
Late-phase BCF indices						
Stumvoll second phase	60 (52, 69)	13	50 (33, 67)	21	54 (46, 63)	13–14
I_120_/I_0_ ratio	60 (52, 67)	15	51 (35, 66)	19	63 (55, 71)	1
CP_120_/CP_0_ ratio	54 (46, 62)	18	56 (41, 71)	13	62 (54, 70)	2–3
ΔI_120_/ΔG_120_ ratio	54 (47, 61)	17	54 (39, 69)	15	55 (48, 63)	8
ΔCP_120_/ΔG_120_ ratio	52 (48, 58)	19	52 (39, 65)	17	55 (47, 62)	12
CIR_120_	65 (57, 73)	8	56 (40, 72)	14	52 (45, 60)	17
Overall insulin secretion indices						
I_AUC_/ G_AUC_ ratio	61 (53, 70)	11	53 (37, 69)	16	52 (44, 61)	18
CP_AUC_/ G_AUC_ ratio	68 (60, 76)	7	63 (48, 78)	11	56 (47, 64)	7

ROC AUCs are expressed in % (95% CI)

‘Rank’ reflects the order of ROC AUC estimates from high to low. Differences between ranked ROC AUCs were not statistically significant

BIGTT-AIR, beta cell function, insulin sensitivity and glucose tolerance testing

ROC AUCs for incident type 2 diabetes revealed estimates higher than 70% for I_30_/I_0_, CP_30_/CP_0_, ΔCP_30_/ΔG_30_ and corrected insulin response at 30 min (CIR_30_), with ROC AUCs ranging between 74% and 81% (Table 4). Restricting the population to individuals who had NGM at baseline did not materially alter the results; the same five BCF indices and CP_0_/G_0_ had ROC AUCs higher than 70% (AUCs 70–84%). For incident prediabetes, the ROC AUCs of all BCF indices were less than 70%.

**Fig. 1:**
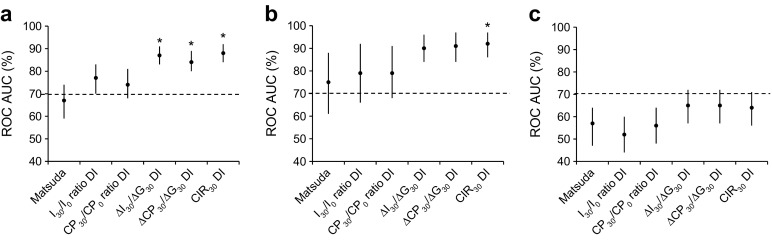
ROC AUCs and corresponding 95% CIs of the oral DIs for: (**a**) discriminatory abilities for incident type 2 diabetes mellitus (T2DM) in at-baseline non-T2DM; (**b**) discriminatory abilities for incident T2DM in at-baseline NGM; and (**c**) discriminatory abilities for incident prediabetes in at-baseline NGM, CODAM study, Maastricht, the Netherlands, 1999–2009. Dashed line, ROC AUC 50%. *p < 0.05 for ROC AUC vs Matsuda index alone

After calculating the DIs, the ROC AUCs for ΔI_30_/ΔG_30_, ΔCP_30_/ΔG_30_ and CIR_30_ had the best discriminatory abilities (Fig. 1; see electronic supplementary material [ESM] Fig. 1 for ROC characteristics). Moreover, for incident type 2 diabetes, these BCF DIs had significantly better abilities than the Matsuda index alone (Fig. 1a).

### Additional analyses

The ability of all BCF indices to cross-sectionally discriminate between glucose metabolism groups was evaluated. ROC AUCs for type 2 diabetes revealed estimates higher than 70% for all early-phase indices, the late-phase index CIR_120_ and the two overall insulin secretion indices (ESM Table 1). The five indices that were ranked highest in the prospective analyses for incident type 2 diabetes, in individuals who were non-diabetic or had NGM at baseline, also showed high ROC AUCs in the cross-sectional analyses, with ROC AUCs in the range 78–88% and 82–92%, respectively. The three DIs that were ranked highest in the longitudinal analyses, ΔI_30_/ΔG_30_, ΔCP_30_/ΔG_30_ and CIR_30_, also had the best ROC AUCs in the cross-sectional analyses (data not shown).

In individuals who were non-diabetic or had NGM at baseline, only the mathematical model variable of beta cell glucose sensitivity was able to discriminate incident type 2 diabetes (ROC AUCs of 78% for both groups). Compared with the simple BCF indices, only the C-peptidogenic index had better discriminatory abilities than beta cell glucose sensitivity for predicting incident type 2 diabetes. ROC AUCs of rate sensitivity and potentiation factor ratio were less than 70% (data not shown).

## Discussion

In this study, for incident prediabetes and type 2 diabetes, the discriminatory ability of available simple OGTT-based indices of BCF was evaluated after a median follow-up period of 7 years. Overall, early-phase indices of BCF, typically based on the insulin response in the first 30 min of the OGTT, performed best.

The better ability of early-phase BCF indices to discriminate glucose metabolism status is in line with previous validation studies, which used the frequently sampled IVGTT or incident type 2 diabetes as reference standards [20, 21]. Moreover, this finding corresponds with an earlier and stronger deterioration of the early-phase secretory response in the pathogenesis of type 2 diabetes [1, 22]. The BCF indices ranked highest in our study also performed well in the evaluations of Hanson et al [20] and Lorenzo et al [21]. In general, discriminatory abilities of BCF indices using C-peptide were better than those using insulin in a similar formula. This can be explained by the fact that insulin concentrations assessed in peripheral blood do not perfectly reflect insulin secretion by the pancreas. Pancreatic beta cells secrete insulin into the portal vein perfusing the liver, where insulin is partially cleared prior to entering the peripheral circulation [2–4]. A more valid estimation of prehepatic insulin secretion can be obtained from C-peptide, which is co-secreted with insulin in equimolar amounts and avoids hepatic degradation [2–4].

For prediabetes, we observed a lower discriminatory ability of the BCF indices than was seen for type 2 diabetes. This probably reflects the gradual deterioration of BCF during the pathogenesis of type 2 diabetes. In prediabetic individuals, insulin secretion is still sufficient to compensate for a certain level of insulin resistance and to maintain glucose levels within the non-diabetic range [2, 23]. In our data, more detailed analyses of the subtypes of prediabetes, i.e. impaired fasting glucose and impaired glucose tolerance, revealed a trend towards higher ROC AUCs for individuals with impaired glucose tolerance relative to those with NGM than for individuals with impaired fasting glucose relative to NGM (data not shown). This is in line with the post-load glucose metabolism of individuals with impaired fasting glucose being normal, reflecting an adequate (compensatory) post-load BCF. Future studies that focus on estimates of insulin secretory function in prediabetes subtypes may provide novel insights into the aspects of BCF covered by the several BCF indices.

In line with previous findings [24, 25], discriminatory abilities of the oral DIs CIR_30_ and ΔI_30_/ΔG_30_ were significantly better than those of the isolated BCF indices in the current study. Moreover, discriminatory abilities of the oral DIs were better than those of the Matsuda index alone, especially the DIs of ΔI_30_/ΔG_30_, ΔCP_30_/ΔG_30_ and CIR_30_. This suggests that ΔI_30_/ΔG_30_, ΔCP_30_/ΔG_30_ and CIR_30_ contributed significantly to the discrimination of glucose metabolism groups, on top of insulin sensitivity. This indicates specific features of BCF irrespective of insulin sensitivity.

ROC analyses of logistic regression outcomes of the BCF indices with the Matsuda index as a covariate (using the probabilities predicted by the regression model) revealed similar results, as presented in Fig. 1 (data not shown). Theoretically, a hyperbolic function between BCF and insulin sensitivity has been suggested as a requirement to apply the DIs. In our data, such a hyperbolic function between any of the BCF indices and a measure of insulin sensitivity (e.g. the Matsuda index, HOMA-IR or the insulin sensitivity index (SI_0,__120_)] was not present [data not shown]). This is in line with some other studies [26, 27], but there are studies that have established a hyperbolic function between BCF and insulin sensitivity [26, 28]. In addition, artificial relations between indices of BCF and insulin sensitivity may arise when these indices are obtained from insulin and glucose concentrations during a single OGTT [26, 29]. In this case, the DIs should be calculated with caution.

We found that the C-peptidogenic index (ΔCP_30_/ΔG_30_) performed better than the mathematical model variables in the present study. However, the good performance of the C-peptidogenic index does not necessarily imply a weakness of the modelling analysis. The reason is that the mathematical model measures specific aspects of BCF (beta cell glucose sensitivity, beta cell potentiation ratio and beta cell rate sensitivity), which together determine glucose metabolism. In contrast, beta cell glucose sensitivity and beta cell rate sensitivity, which have been shown to be relatively independent and both potentially important predictors of type 2 diabetes, are lumped together in the C-peptidogenic index [30]. This may confer on the empirical index a relative advantage in terms of predictive power.

The present study benefits from the comparison of all available BCF indices in one study population, which includes individuals with NGM, prediabetes and newly diagnosed type 2 diabetes. Moreover, the study has a longitudinal set-up with 7 years of follow-up. Furthermore, the assessment of plasma C-peptide concentrations enabled comparative analyses between BCF indices based on insulin and C-peptide concentrations. Some limitations also merit discussion. First, glucose metabolism status, BCF and insulin sensitivity were all obtained from a single OGTT [31]. We aimed to minimise autocorrelation between BCF and insulin sensitivity by using the Matsuda index, which is based on fasting and mean plasma insulin and glucose concentrations during the OGTT, rather than on concentrations at specific time points only. Although our interest was in discrimination, and not in association estimates, correlations between the BCF indices and the Matsuda index were very weak overall, and additional multicollinearity diagnostics (i.e. the variance inflation factor) were negative. Finally, CODAM participants have a higher risk of type 2 diabetes than the general population, so generalisability might be limited. However, as the differences in BCF between non-diabetic and type 2 diabetic individuals in the present study population may be smaller than in the general population, the discriminatory abilities of the BCF indices in the general population may be larger.

In summary, early-phase BCF indices obtained from an OGTT revealed those with the best abilities to discriminate glucose metabolism status. Overall, the C-peptidogenic index (ΔCP_30_/ΔG_30_) had the best discriminatory ability. If the degree of insulin sensitivity is taken into account by calculating the DIs, the insulinogenic index (ΔI_30_/ΔG_30_), the C-peptidogenic index (ΔCP_30_/ΔG_30_) and CIR_30_ had the highest ability to discriminate glucose metabolism status. Based on our results, we recommend the C-peptidogenic index (ΔCP_30_/ΔG_30_) for assessing BCF in epidemiological studies. However, the preferred combination of BCF indices reflecting different aspects of insulin secretory function should be determined in view of the specific objectives and hypotheses of the study.

## Electronic supplementary material

Below is the link to the electronic supplementary material.

ESM (PDF 396 kb)

## Abbreviations

BCF: Beta cell function
CIR_*x*_: Corrected insulin response measured *x* min after the start of the OGTT
CODAM: Cohort on Diabetes and Atherosclerosis Maastricht
CP_*x*_: C-peptide response measured *x* min after the start of the OGTT
DI: Disposition index
G_*x*_: Glucose response measured *x* min after the start of the OGTT
I_*x*_: Insulin response measured *x* min after the start of the OGTT
IQR: Interquartile range
NGM: Normal glucose metabolism
ROC: Receiver operating characteristics
SQUASH: Short Questionnaire to Assess Health-enhancing Physical Activity
