# Supplementary material for: Discriminatory ability of simple OGTT-based beta cell function indices for prediction of prediabetes and type 2 diabetes: the CODAM study
Source: Diabetologia. 2016 Dec 8;60(3):432–41. doi: 10.1007/s00125-016-4165-3 (PMC6518926; doi:10.1007/s00125-016-4165-3)
Supplement: Supplementary file 1 — (PDF 396 kb) [file 125_2016_4165_MOESM1_ESM.pdf]

ESM table 1: Discrimination Between Individuals with Prevalent T2DM (n=60) or Prediabetes (n=122) and Either NGM (n=294) or Non-diabetes (n=416) by BCF-indices

	Non-T2DM versus T2DM	Rank	T2DM versus NGM	Rank	Prediabetes versus NGM	Rank
Fasting BCF-indices						
HOMA B1	60 (52, 68)	16	61 (53, 69)	15	52 (45, 59)	21
HOMA B2	65 (57, 73)	13-14	67 (58, 75)	14	53 (46, 59)	20
I_0/G_0 ratio	59 (50, 67)	18	61 (52, 70)	16	57 (51, 64)	11
CP_0/G_0 ratio	58 (51, 65)	20	60 (52, 68)	17	56 (50, 63)	14
Early-phase BCF-indices						
I_{30}/I_0 ratio	85 (80, 90)	3	89 (85, 94)	3	67 (61, 72)	6
CP_{30}/CP_0 ratio	88 (83, 92)	1	92 (88, 95)	1	71 (66, 76)	2
$\Delta I_{30}/\Delta G_{30}$ ratio	79 (73, 84)	6	83 (77, 88)	6	66 (60, 72)	7
$\Delta I_{30}/G_{30}$ ratio	78 (72, 84)	7-8	82 (76, 88)	7-8	57 (51, 63)	12-13
$\Delta CP_{30}/\Delta G_{30}$ ratio	84 (79, 89)	4	89 (84, 93)	4	73 (67, 78)	1
CIR_{30}	87 (82, 91)	2	91 (87, 95)	2	68 (63, 73)	5
Stumvoll early-phase	68 (60, 77)	12	71 (63, 79)	12	54 (48, 61)	15-16
$BIGTT\text{-}AIR_{0.30.120}$	74 (67, 81)	9	78 (71, 85)	9	57 (51, 63)	12-13
$BIGTT\text{-}AIR_{0.60.120}$	73 (66, 80)	10	76 (69, 84)	10	62 (55, 68)	8
Late-phase BCF-indices						
Stumvoll second phase	65 (57, 73)	13-14	67 (58, 76)	13	54 (48, 61)	15-16
I_{120}/I_0 ratio	54 (46, 61)	21	55 (48, 61)	21	69 (64, 75)	3-4
CP_{120}/CP_0 ratio	60 (51, 68)	17	57 (48, 65)	19	53 (47, 58)	18-19
$\Delta I_{120}/\Delta G_{120}$ ratio	58 (52, 64)	19	55 (49, 61)	20	54 (48, 59)	17
$\Delta CP_{120}/\Delta G_{120}$ ratio	62 (57, 68)	15	58 (52, 64)	18	53 (47, 58)	18-19
CIR_{120}	82 (78, 87)	5	84 (79, 88)	5	69 (64, 75)	3-4
Overall BCF-indices						
I_{AUC} / G_{AUC} ratio	71 (64, 78)	11	73 (66, 81)	11	60 (54, 66)	9
CP_{AUC}/ G_{AUC} ratio	78 (72, 84)	7-8	82 (76, 88)	7-8	59 (53, 65)	10

T2DM, type 2 diabetes; NGM, normal glucose metabolism; Non-T2DM = normal glucose metabolism + prediabetes
Values represent ROC AUC in % (95%-confidence interval).
'Rank' reflects the order of ROC AUC estimates from high (1) to low (20). Differences between consecutively ranked ROC AUCs were not statistically significant.

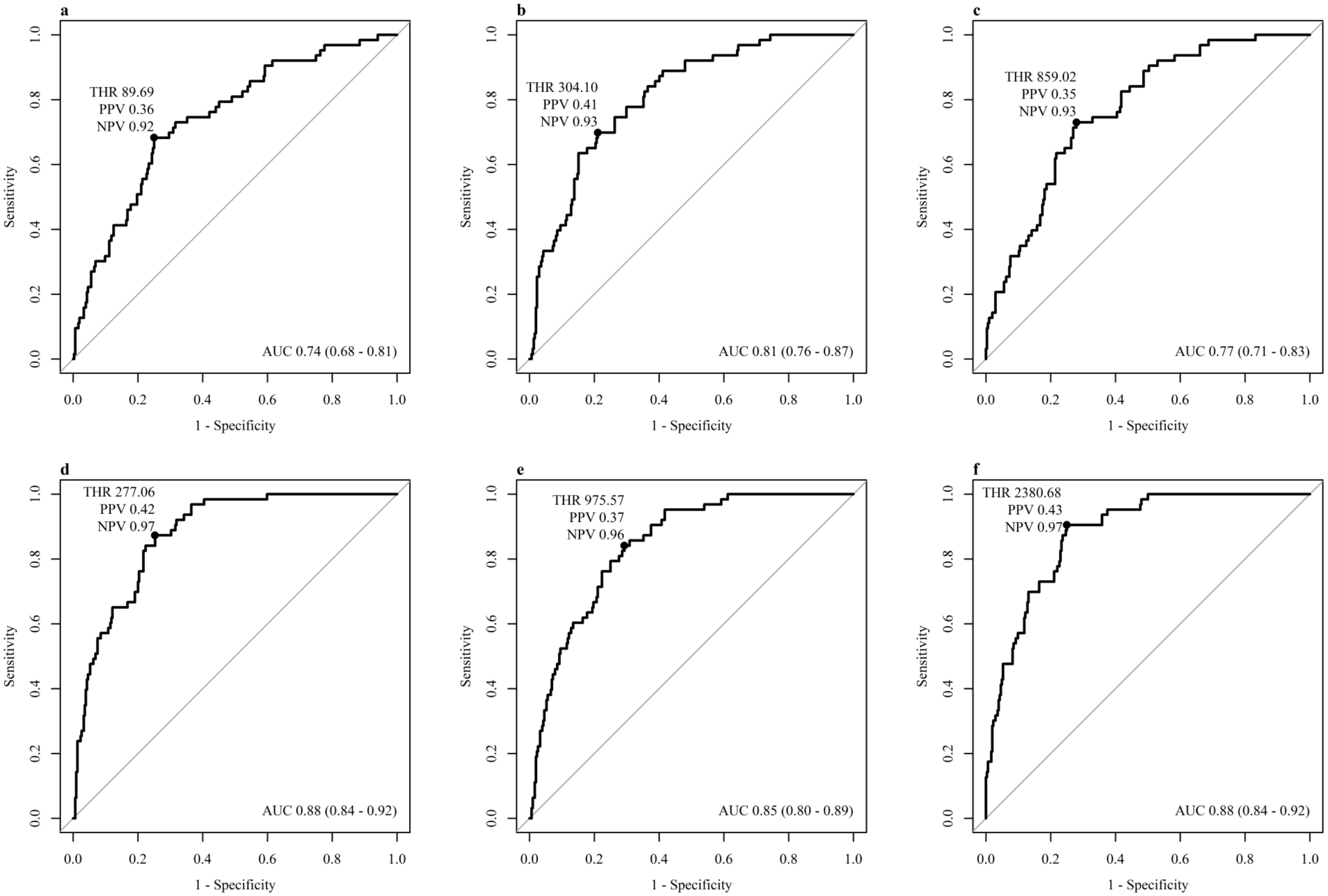

ESM figure 1: ROC curves and characteristics of the three BCF indices and corresponding disposition indices that consistently ranked highest:

a) $\Delta I_{30}/\Delta G_{30}$

b) $\Delta CP_{30}/\Delta G_{30}$

c) CIR_{30}

d) $\Delta I_{30}/\Delta G_{30}$ DI

e) $\Delta CP_{30}/\Delta G_{30}$ DI

f) CIR_{30} DI

AUC, Area under the Receiver Operating Curve (95% CI); THR, threshold value for the specific BCF index (Youden index); PPV, positive predicted value; NPV, negative predicted value

The *point on the ROC curve represents the THR.*
